# Phase Property of Envelope-Tracking EEG Response Is Preserved in Patients with Disorders of Consciousness

**DOI:** 10.1523/ENEURO.0130-23.2023

**Published:** 2023-08-09

**Authors:** Ziting Jia, Chuan Xu, Jingqi Li, Jian Gao, Nai Ding, Benyan Luo, Jiajie Zou

**Affiliations:** 1 The Second Hospital, Cheeloo College of Medicine, Shandong University, Jinan 250033, China; 2 Key Laboratory for Biomedical Engineering of Ministry of Education, College of Biomedical Engineering and Instrument Sciences, Zhejiang University, Hangzhou 310027, China; 3 Department of Neurology, First Affiliated Hospital, School of Medicine, Zhejiang University, Hangzhou 310003, China; 4 Department of Rehabilitation, Hangzhou Mingzhou Brain Rehabilitation Hospital, Hangzhou 311215, China; 5 Department of Neurology, Sir Run Run Shaw Hospital, Zhejiang University School of Medicine, Hangzhou 310019, China

**Keywords:** envelope tracking, neural entrainment, phase coherence, speech

## Abstract

When listening to speech, the low-frequency cortical response below 10 Hz can track the speech envelope. Previous studies have demonstrated that the phase lag between speech envelope and cortical response can reflect the mechanism by which the envelope-tracking response is generated. Here, we analyze whether the mechanism to generate the envelope-tracking response is modulated by the level of consciousness, by studying how the stimulus-response phase lag is modulated by the disorder of consciousness (DoC). It is observed that DoC patients in general show less reliable neural tracking of speech. Nevertheless, the stimulus-response phase lag changes linearly with frequency between 3.5 and 8 Hz, for DoC patients who show reliable cortical tracking to speech, regardless of the consciousness state. The mean phase lag is also consistent across these DoC patients. These results suggest that the envelope-tracking response to speech can be generated by an automatic process that is barely modulated by the consciousness state.

## Significance Statement

During speech listening, a prominent cortical response is the speech envelope-tracking activity. In the frequency domain, the two fundamental characteristics of envelope-tracking activity are power and phase. Recent studies have demonstrated that the phase property of envelope-tracking activity can reveal its underlying generation mechanism. In this study, we investigate whether this generation mechanism is modulated by the state of consciousness. We introduce healthy individuals and patients with disorders of consciousness. Results demonstrate that the stimulus-response phase lag changes linearly with frequency for both healthy individuals and patients who exhibit reliable neural tracking of the speech envelope. Thus, envelope-tracking activity is generated through an automatic process, which is not strongly modulated by the state of consciousness.

## Introduction

When listening to speech, a prominent cortical response is the response that tracks the speech envelope, i.e., low-frequency fluctuation in sound intensity (Lalor et al., 2009; Ding and Simon, 2012a; Wang et al., 2012; Peelle et al., 2013; Doelling et al., 2014; Harding et al., 2019). The speech envelope is critical for speech intelligibility and prevalent in natural sounds (Drullman et al., 1994; Shannon et al., 1995; Shamma, 2001; Elliott and Theunissen, 2009; Ding et al., 2017). The speech envelope is extracted from the sound input in the auditory periphery (Yang et al., 1992; Shamma, 2001), and the low-frequency components of the speech envelope is amplified through the auditory processing pathway (Sharpee et al., 2011). Intracranial recordings have revealed that, in cortex, the low-frequency envelope-tracking neural response is observed both in auditory cortex and in other widely distributed temporal and frontal areas (E.M. Zion Golumbic et al., 2013b). Since the envelope-tracking response can be reliably measured from individuals and is an auditory response that receives the modulation from higher-order cortical areas, it has been applied widely to study auditory processing in special populations (Braiman et al., 2018; Xu et al., 2021).

The physiological interpretation of the envelope-tracking response, however, has been controversial. On the one hand, since the envelope-tracking response is phase locked to the speech input, it may purely reflect bottom-up sensory evoked responses (Steinschneider et al., 2013). Some studies further suggest that the bottom-up sensory mechanism generating the envelope-tracking response can be well approximated by a linear system (Lalor et al., 2009; Ding and Simon, 2012a,b). In other words, the envelope-tracking response is well approximated as a superposition of the neural responses independently evoked by auditory features. This hypothesis is referred to as the evoked response hypothesis. On the other hand, the envelope-tracking response is strongly modulated by top-down attention (Ding and Simon, 2012b; Power et al., 2012; O’Sullivan et al., 2015), and is influenced by language proficiency (Zou et al., 2019), prior information (Wang et al., 2019), and multisensory input (E. Zion Golumbic et al., 2013a; Crosse et al., 2016). The strong top-down and multisensory modulation effects lead to the hypothesis that the envelope-tracking response reflects a modulation signal from higher-level cortical areas, e.g., ventral prefrontal cortex (Schroeder et al., 2008; van Atteveldt et al., 2014; Giordano et al., 2017). Specifically, the modulation signal resets the phase of ongoing oscillations in, e.g., auditory cortex, so that the ongoing oscillations track the speech envelope. For this hypothesis, referred to as the oscillation phase resetting hypothesis, the phase of neural oscillations is an index for neural excitability (Lakatos et al., 2013). Consequently, when neural oscillations show a phase that indexes high neural excitability, the sensory input will be better encoded (Jeremy et al., 2011; van Atteveldt et al., 2014) and the high excitability phase is often referred to as the optimal phase (Schroeder et al., 2008; Henry and Obleser, 2012; Ng et al., 2012).

In the frequency domain, the neural response tracking speech envelope can be decomposed into response power and response phase at each frequency (Luo and Poeppel, 2007). Recent studies have shown that the response phase carries important information about how the envelope-tracking response is generated (Doelling et al., 2019; Zou et al., 2021). Suppose the envelope-tracking response is generated by purely bottom-up mechanisms: the auditory periphery extracts the speech envelope and envelope-tracking neural activity is transmitted from auditory nerves to auditory cortex. For such a purely bottom-up mechanism, neural activity in cortex tracks the speech envelope with a constant delay that corresponds to the neuronal transmission time and the time to generate large-scale synchronized cortical responses. In this condition, in the frequency domain, the stimulus-response phase lag is a linear function of response frequency. Evidence for such linear-phase property is previously observed in healthy individuals who passively listen to speech (Zou et al., 2021).

In contrast, if the mechanisms generating the envelope-tracking response engage complex interactions between multiple cortical or subcortical areas, as is emphasized by the oscillation phase resetting hypothesis, the cortical response will not just be a delayed version of the speech envelope. Consequently, in the frequency domain, the stimulus-response phase lag will not reduce to a simple linear function in general. In particular, when healthy individuals actively listen to music, it has been shown that the stimulus-response phase lag is around 0 degree across frequencies (Doelling et al., 2019), consistent with the hypothesis that an optimal, i.e., high-excitability, phase is always aligned to the stimulus regardless of its presentation frequency (Schroeder et al., 2008; Doelling et al., 2019).

For healthy individuals, the speech envelope-tracking response is influenced by both the bottom-up speech input and top-down feedback from higher-level cognitive systems. Therefore, it is challenging to tease apart whether the linear-phase property and latency of envelope-tracking activity is determined by bottom-up processes or an interaction between bottom-up sensory encoding and top-down feedback. Here, we investigate whether the response phase property and response latency are preserved when top-down cognitive modulation diminishes. To reduce top-down neural modulation, we test patients with disorder of consciousness (DoC), which is caused by extensive or focal injuries to neural tissues that lead to the large-scale dysfunctions of the central nervous system (Giacino et al., 2014). DoC patients can be further divided into groups who have different levels of consciousness, e.g., patients in the unresponsive wakefulness syndrome (UWS)/vegetative state (VS; Ashwal, 1994; Laureys et al., 2010), patients in the minimal conscious state (MCS; Giacino et al., 2002), and patients emerged from a minimally conscious state (EMCS). Here, we analyze whether the phase properties of the envelope-tracking response are influenced by the DoC, who can have preserved bottom-up auditory processing (Giacino et al., 2014; Beukema et al., 2016) but their top-down cognitive control is severely impaired (Daniel et al., 2016; Giacino et al., 2018). If the envelope-tracking response primarily reflects bottom-up processing, its phase property can be preserved in DoC patients. In contrast, if the envelope-tracking response critically relies on top-down neural modulation, its phase properties should be altered by the DoC.

## Materials and Methods

### Participants

This study analyzed the phase properties of envelope-tracking neural activity based on an EEG dataset that included healthy individuals, MCS patients, and UWS patients (Xu et al., 2021). In addition, following the same experimental procedure in Xu et al. (2021), this study also collected data from EMCS patients. In total, data from 56 participants were reported (16 UWS: 12 males, 56.81 ± 12.75 years; 15 MCS: 14 males, 49.07 ± 16.55 years; 9 EMCS: 9 males, 50.78 ± 13.64 years; 16 healthy individuals, 5 males; 54.25 ± 9.88 years). There was no significant age difference between healthy individuals and any of the three patient populations (one-way ANOVA, *p *=* *0.339). No significant difference in brain injury duration was observed between the three patient populations (one-way ANOVA, *p *=* *0.224). The study was approved by the Ethical Committee of the First Affiliated Hospital of Zhejiang University, and by Hangzhou Mingzhou Brain Rehabilitation Hospital. Written informed consent was provided by participants or their legal surrogates for the experiments and for publication of their individual details in this study.

### Stimuli and experimental procedures

Participants were exposed to natural speech through headphones in a patient room. The stimulus included two chapters from Cixin Liu’s novel, *The Supernova Era* (Chapter 16: “Fun country” and Chapter 18: “Sweet dream period”). The speech was narrated in Mandarin Chinese by a female speaker and digitized at a 48-kHz sampling rate. The speech was clear and highly intelligible. The duration of the two chapters were 34 and 25 min, respectively, and responses to the two chapters were concatenated in analyses.

EEG response were recorded while participants listened to speech. The experiment was conducted in 2 d, and the spoken narrative was presented once on each day. The DoC participants had their eyes open at the beginning of each day’s experiment. Both healthy individuals and EMCS patients were instructed to remain still throughout the experiment. No additional tasks or instructions were given.

### EEG recording and preprocessing

EEG signals were recorded using a 64-electrodes BrainCap (Brain Products GmbH) following the international 10–20 system. One of these electrodes was positioned under the right eye to record electrooculogram (EOG). EEG signals were initially referenced online to FCz but were later referenced offline to a common average reference. To remove line noise, a 50-Hz notch filter was applied, along with a low-pass antialiasing filter with a 70-Hz cutoff and a high-pass filter with a 0.3-Hz cutoff to prevent slow drifts (both eighth order zeros-phase Butterworth filters). Signals were sampled at 1 kHz and processed according to the procedure detailed previously (Zou et al., 2019). All preprocessing and analysis were performed using MATLAB software (The MathWorks).

EEG recordings underwent low-pass filtering below 50 Hz using a zero-phase anti-aliasing FIR filter (implemented using a 200-ms Kaiser window) and down-sampled to 100 Hz. EOG artifacts were eliminated through regression based on the least-squares method. The same as in previous studies (Ding and Simon, 2012a,b), the speech response was averaged over the two representations on both recording days to enhance the signal-to-noise ratio.

The speech envelope was obtained by applying full-wave rectification to the speech (Zou et al., 2021) and low-pass filtering it below 50 Hz using a zero-phase anti-aliasing FIR filter (implemented using a 200-ms Kaiser window). The envelope was further down-sampled to 100 Hz.

### Phase extraction and phase coherence

**Figure 1.**
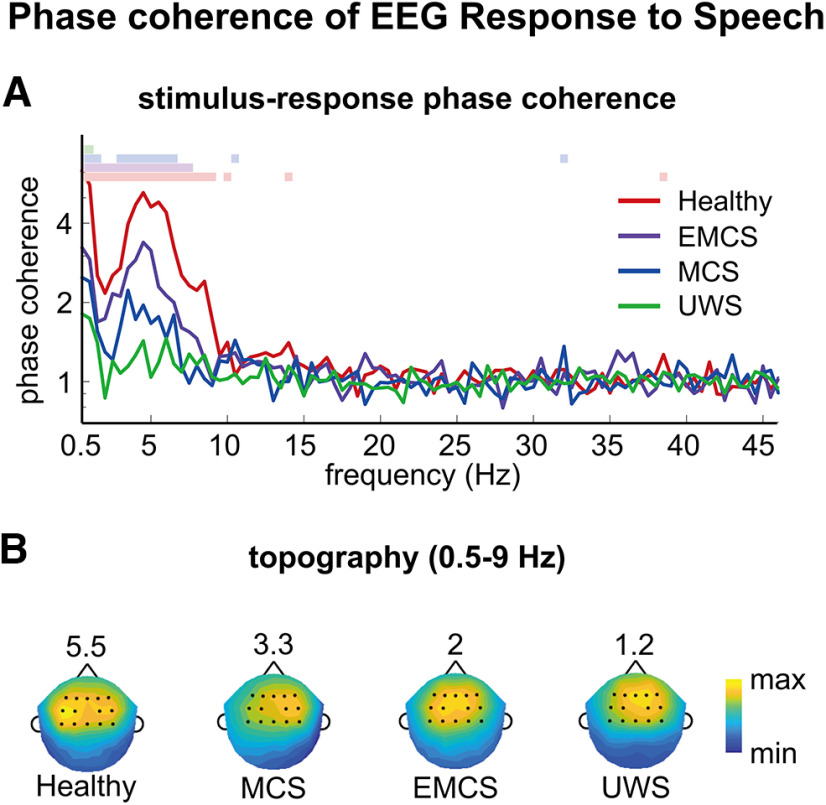
Phase coherence spectrum. ***A***, The phase coherence spectrum shows how precisely the response is synchronized to the stimulus. The colored lines on top denote frequency bins in which the phase coherence is significantly higher than chance (*p *<* *0.01, permutation test, FDR corrected). Stimulus-response phase synchronization is significantly reliable below ∼9 Hz. ***B***, Topography of phase coherence. To better illustrate the spatial distribution, the phase coherence is separately normalized in each plot by dividing by the 95th percentile of phase coherence across electrodes, and the values of the 95th percentile is shown on top of each plot. The dark dots represent the 14 centro-frontal electrodes chosen for subsequent phase analysis.

To assess the stimulus-response phase lag, both the speech envelope and EEG response were converted into the frequency domain, with each electrode being independently analyzed. Specifically, the speech envelope and EEG response were divided into nonoverlapping 2-s time bins and subsequently transformed into the frequency domain using the discrete Fourier transform (DFT) via the fast Fourier transform (FFT) algorithm. The response phase (*α_ft_*) and stimulus phase (*β_ft_*) in frequency bin *f* and time bin *t* were used to determine the stimulus-response phase lag *θ_ft_* as *α_ft_* − *β_ft_*. The coherence of the phase lag across time bins, referred to as the cerebro-acoustic phase coherence (Peelle et al., 2013), was computed using this equation:

$$C(f)=\frac{{\left(\sum_{t=1}^{T}cos(\theta_{ft})\right)}^{2}+{\left(\sum_{t=1}^{T}sin(\theta_{ft})\right)}^{2}}{T^{2}},$$

where *C*(*f*) was the phase coherence in frequency bin *f*. Greater phase coherence implies that the response phase more precisely follows the stimulus phase. With phase coherence being strongest in central-frontal electrodes for all participant groups (Fig. 1*B*), 14 centro-frontal electrodes (i.e., Fz, F1, F2, F3, F4, FC1, FC2, FC3, FC4, Cz, C1, C2, C3, and C4) were used to compare the phase coherence value across populations and examine the phase-frequency relationship.

### Phase-frequency relationship

The stimulus-response phase lag at frequency *f*, denoted as *θ_f_*, was computed by averaging *θ_ft_* across all 2-s time bins using the circular mean (Fisher, 1993). Group delay is characterized based on the first-order derivative of the stimulus-response phase lag across frequency, i.e., *d*(*f*) = (*θ*(*f*) − *θ*(*f* + Δ*f*))/2πΔ*f* (Oppenheim et al., 1997). The group delay was computed by unwrapping the phase lag, calculating the difference between adjacent frequency bins, and dividing the difference by π. The mean phase difference was computed as 2(*θ*(*f*) − *θ*(*f* + Δ*f*) + *θ*(*f *+* *2Δ*f*) − *θ*(*f *+* *3Δ*f*) + . . . + *θ*(*f* + (*N *−* *1)Δ*f*) − *θ*(*f* + *N*Δ*f*))/Δ*f*/(*N *−* *1). The mean phase was transformed into a group delay by dividing it by π.

To assess the linearity of the phase-frequency curve, the absolute value of its second-order derivative was calculated using the equation: *d*_2_(*f*) = *|θ*(*f*) +*θ*(*f +*2Δ*f*)* *−* *2*θ*(*f* + Δ*f*)|. A second-order derivative *d*_2_(*f*) of 0 would indicate a linear change in phase lag with frequency. Thus, *d*_2_(*f*) reflects the linearity of the phase-frequency curve, where lower *d*_2_(*f*) values indicate a more linear curve.

As the phase-frequency curve exhibited a near-linear relationship between 3.5 and 8 Hz, a linear function was used to approximate the actual phase-frequency curve within this range: *θ_L_*(*f*) = *kf* + *b*, for 3.5 ≤ *f* ≤ 8. The slope parameter *k* and the intercept parameter *b* were fitted separately for each participant population using the least-squares method.

### Statistics

In order to assess whether the phase coherence at a specific frequency was significantly greater than chance, we employed a permutation approach to estimate the chance-level phase coherence (Peelle et al., 2013; Harding et al., 2019). After the speech envelope and EEG response were divided into 2-s time bins, the time bins for the speech envelope were shuffled, resulting in a random pairing of the envelope and response. Subsequently, we computed the phase coherence for the phase lag between the response and the randomly paired speech envelope. This process was conducted 5000 times, yielding 5000 chance-level phase coherence. For the significance tests in Figure 1*A*, we computed the averaged phase coherence value across electrodes and participants in each population, for both the actual phase coherence and the 5000 chance-level phase coherence. The significance level of the phase coherence at a specific frequency was (*N *+* *1)/5001, if it was lower than *N* out of the 5000 chance-level coherence at that frequency (one-sided comparison).

A similar procedure was used to determine the chance-level second-order derivative of the phase-frequency curve. The second-order derivative was significantly nearer to 0 than chance, with the significance level being (*N *+* *1)/5001, if it was greater than *N* of the 5000 chance-level values in terms of the absolute value (one-sided comparison).

## Results

The current study aimed to analyze whether the phase lag between speech envelope and cortical response was a linear function of frequency. A prerequisite of the analysis is that the stimulus-response phase lag is reliably measured. Therefore, we first identified which frequency bands and EEG electrodes exhibited reliable phase synchronization between neural response and speech envelope. We computed the coherence of the stimulus-response phase lag for each electrode in each frequency bin separately, and the results in Figure 1*A* were averaged across all electrodes. Significant phase coherence was observed in at least one frequency bin below 9 Hz for all participant populations. The topography of the low-frequency neural responses (<9 Hz) showed a centro-frontal distribution for all 4 groups of participants (Fig. 1*B*). Therefore, we selected 14 centro-frontal channels for further analyses.

**Figure 2.**
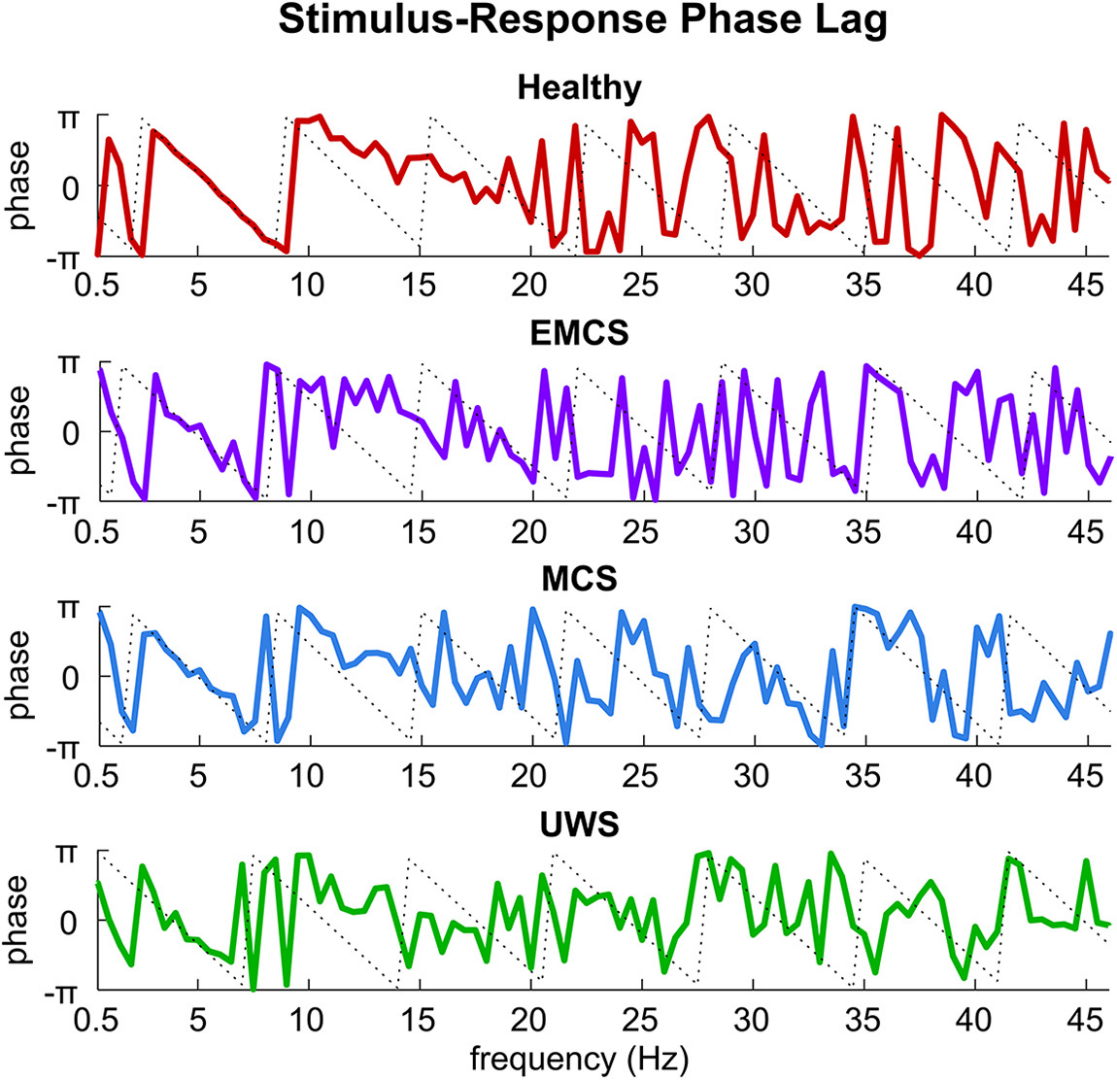
Phase-frequency curve. The phase-frequency curve shows the stimulus-response phase lag as a function of frequency. The phase lag appears to linearly decrease over frequency in a frequency range between 3.5 and 8 Hz. The dotted lines are fitted based on the phase lag between 3.5 and 8 Hz.

**Figure 3.**
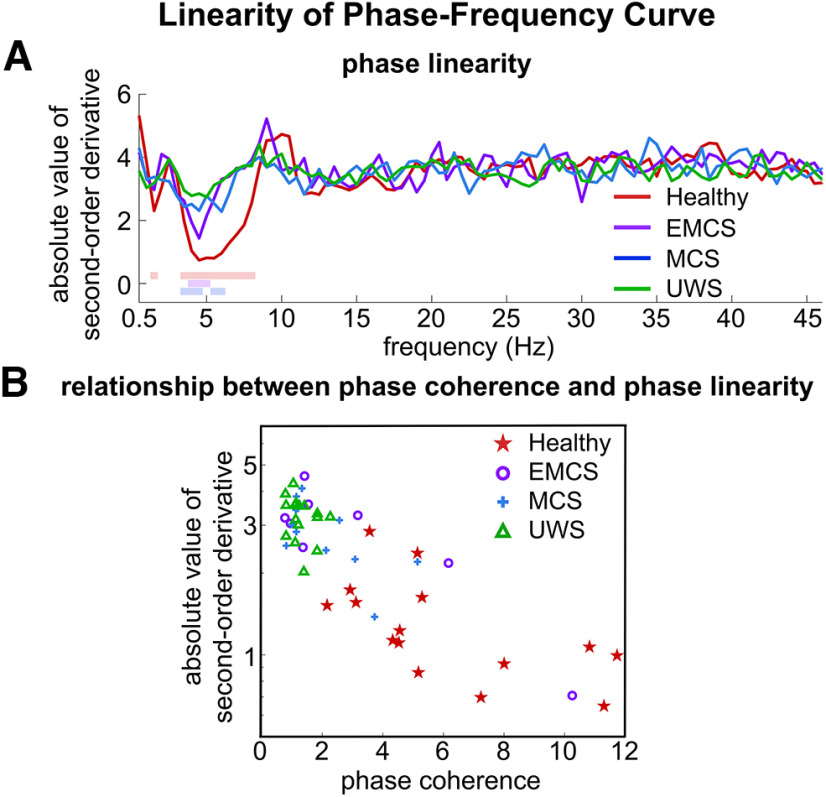
Linearity of the phase-frequency curve. ***A***, The second-order derivative of the phase-frequency curve is used to quantify the linearity of the phase-frequency curve. The second-order derivative is 0 if the stimulus-response phase lag changes linearly with frequency. The colored lines on top denote the frequency bins in which the absolute value of the second-order derivative is significantly closer to 0 than chance (*p* < 0.01, permutation test, FDR corrected). ***B***, The relationship between phase coherence and absolute value of second-order derivative of the phase-frequency curve. The phase coherence and the absolute value of second-order derivative are both averaged between 3.5 and 8 Hz. Participants with higher phase coherence generally show lower absolute value of second order derivatives, i.e., better linearity.

We next investigated how the stimulus-response phase lag varied with frequency. For healthy individuals, the phase lag appeared to change linearly with frequency in the range where the phase coherence exceeded chance levels (Fig. 2*A*). The linearity of the phase-frequency curve was evaluated using its second-order derivative. For a linear function, the second-order derivative equaled 0. As shown in Figure 3*A*, the absolute value of second-order derivative of the phase-frequency curve was significantly closer to 0, i.e., lower than chance, between 3.5 and 8 Hz for healthy individuals (*p *<* *0.01, permutation test, FDR corrected), suggesting a linear phase-frequency function. A straight line was used to fit this linear trend between 3.5 and 8 Hz and was shown by the dotted gray line in Figure 2.

**Figure 4.**
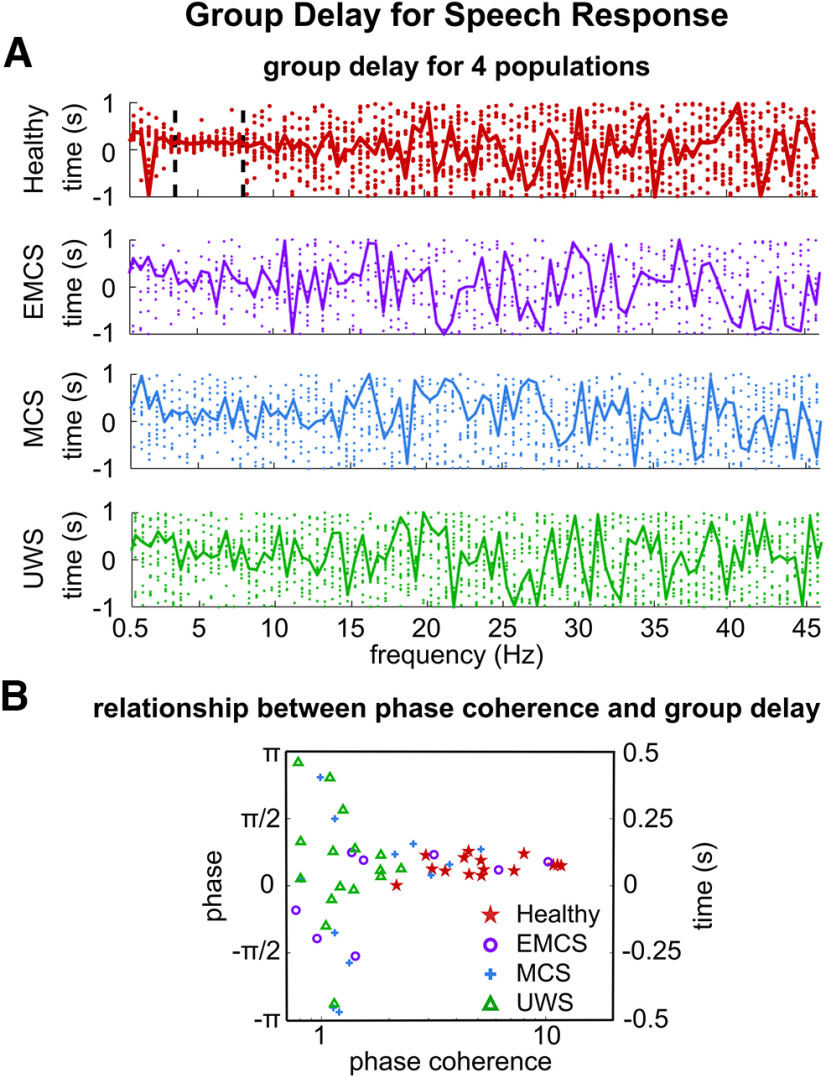
Group delay of the speech response. ***A***, Group delay for four populations. Each dot denotes a participant. Between 3.5 and 8 Hz, the group delay is consistent across healthy individuals, but less consistent for the DoC patients. ***B***, The relationship between group delay and phase coherence for individuals. The *x*-axis is the phase coherence averaged between 3.5 and 8 Hz. The *y*-axis on the left is the mean phase difference between neighboring frequency bins, and the *y*-axis on the right shows the group delay. Participants with higher phase coherence generally show consistent group delay.

The DoC patients showed a similar linear trend in the same frequency range (Fig. 2, lower three panels), although the curves were noisier because of the lower phase coherence (Fig. 1*A*). As shown in Figure 3*A*, for the EMCS and MCS patients, the second-order derivative of the phase-frequency curve was also significantly closer to 0 in some frequency bins between 3.5 and 8 Hz (*p *<* *0.01, permutation test, FDR corrected). For the UWS patients, the second-order derivative showed a similar trend between 3.5 and 8 Hz, but the trend was not significant. It was observed that the phase-frequency curve tended to be more linear for participants who showed higher phase coherence: when the absolute value of the second-order derivative was averaged between 3.5 and 8 Hz, it correlated with the individual phase coherence averaged over the same frequency range (*R* = −0.767, *p *=* *5 × 10^−11^, two-tailed Student’s *t* test; Fig. 3*B*).

Based on the systems theory, the group delay is proportional to the first-order derivative of the phase-frequency function, reflecting how quickly a change in the stimulus is reflected in the response (Oppenheim et al., 1997). Based on the linear fit in Figure 2, the mean group delay between 3.5 and 8 Hz was 152, 147, 152, and 146 ms, for the healthy individuals, EMCS, MCS, and UWS patients, respectively. The group delay at each frequency was shown in Figure 4*A* for all four populations. Healthy individuals showed consistent group delay between 3.5 and 8 Hz (Fig. 4*A*, upper panel). For the DoC patients, the group delay appeared to have larger individual differences (Fig. 4*A*, lower three panels). The large individual difference could be attributed to at least two factors. First, different DoC patients had difference response latency. Second, the stimulus-response phase lag was not reliable. For an extreme case, if the neural response was not synchronized to the stimulus, the group delay would be completely random for each participant. To distinguish these two possibilities, we analyzed the relationship between group delay and the mean phase coherence between 3.5 and 8 Hz for individual participants (Fig. 4*B*). It was observed that participants showing higher phase coherence tended to have similar group delay: the absolute difference between individual group delay and the mean group delay over participants was negatively correlated with individual phase coherence averaged over 3.5 and 8 Hz (*R* = −0.467, *p *=* *6 × 10^−4^, two-tailed Student’s *t* test). This result suggested common group delay for individuals who show reliable phase coherence.

## Discussion

The phase-frequency curve is a fundamental character of a system, and here we analyze how the phase-frequency curve of speech envelope-tracking response is modulated by the state of consciousness. The stimulus-response phase coherence is reduced by the DoC, but it is demonstrated that the linear-phase property can be observed in both healthy individuals and in EMCS/MCS/UWS patients who exhibit reliable neural synchronization to speech. This result indicates the phase property of envelope-tracking neural activity is not strongly modulated by the state of consciousness, in favor of the evoked response hypothesis (Ding and Simon, 2012b; Power et al., 2012; O’Sullivan et al., 2015; Zou et al., 2021).

What kind of systems can show a linear-phase property? The simplest form of such a system is a delay system, for which the response is simply the stimulus but delayed. Suppose the delay of the system is *T*. When the stimulus to the system is a sinusoid at *f* Hz, the response is also an *f*-Hz sinusoid delayed by *T*. A delay *T* corresponds to a 2π*Tf* phase shift of the *f*-Hz sinusoid. Therefore, the stimulus-response phase lag is 2π*Tf*, a linear function of *f*. The delay *T*, in this case, is the same as the group delay of the system. More generally, based on the systems theory, if the stimulus-response phase lag changes linearly across frequency, it indicates that the evoked response has a finite duration and has a symmetric waveform centered at the group delay (Oppenheim et al., 1997).

The current study reveals that between 3.5 and 8 Hz, the envelope-tracking response exhibits the linear-phase property, indicating the EEG response resembles the speech envelope but delayed. More importantly, such linear-phase property and even the group delay is largely unchanged in DoC patients as long as they show reliable envelope-tracking activity. In other words, DoC may result in less precise phase synchronization to speech envelope but does not strongly modulate the phase lag between stimulus and response. The reduced precision in phase synchronization may also be the consequence of the reduction of response amplitude: the envelope-tracking response and spontaneous neural activity are both recorded and the ratio between these two components can contribute to the phase synchronization precision.

These results suggest that cortical areas impaired in DoC patients may disable the envelope-tracking response. Nevertheless, in some patients, the envelope-tracking response is not disabled and the properties of the envelope-tracking response are largely maintained. In general, these results are consistent with previous findings that some DoC patients may have preserved bottom-up auditory responses although the response is less reliable than healthy individuals (Fischer et al., 2000; Qin et al., 2008; Gui et al., 2020; Xu et al., 2023).

The frequency range in which the linear phase property is observed, i.e., 3.5–8 Hz, also coincides with the frequency range in which the phase coherence is relatively high. Therefore, it is possible that the phase linearity is lower outside the 3.5- to 8-Hz range since the stimulus-response phase lag is less reliable outside that frequency range. Previous studies have consistently shown that cortical phase locking to speech significantly decreases above ∼8 Hz (e.g., Luo and Poeppel, 2007; Ding and Simon, 2012b), which is potentially attributable to the lack of high-frequency modulations in speech (Ding et al., 2017). Below 8 Hz, the stimulus-response phase coherence is higher than chance until the lowest frequency being analyzed, i.e., 0.5 Hz. In this frequency range, the phase coherence spectrum shows a bimodal pattern, with one peak between 3.5 and 8 Hz and another peak below 1 Hz. In other words, the phase coherence spectrum seems to have a dip around 2 Hz and a similar trend has been observed in previous studies (Koskinen and Seppä, 2014; Bourguignon et al., 2020). Although the mechanism underlying the 2-Hz dip remain unclear, it is possible that it marks a transition in the neural encoding scheme.

Together with a number of previous studies (Lalor et al., 2009; Ding and Simon, 2012a; Zou et al., 2021), the current study suggests that the neural mechanisms generating envelope-tracking neural activity can be well approximated as a linear system. This linear-system view, however, does not suggest that top-down factors, such as attention, cannot modulate envelope-tracking activity. Instead, many studies that analyze attention modulation of envelope-tracking activity model the envelope-tracking response using a linear system, e.g., using the temporal response function (TRF) approach (Lalor et al., 2009; Ding and Simon, 2012a; Brodbeck et al., 2018), and these studies show that the response gain can be enhanced by selective attention (Ding and Simon, 2012b; Mesgarani and Chang, 2012; E.M. Zion Golumbic et al., 2013b). On top of the response gain change, it is also possible that more active speech processing can engage more sophisticated mechanisms in line with the oscillation phase-resetting hypothesis. This possibility, however, has to be addressed by future studies. In the current study, speech is presented in a quiet environment and previous studies have shown attention only minimally modulate the envelope-tracking response (Kong et al., 2014; Ding et al., 2018; Lu et al., 2023).

In summary, the current results suggest that the neural generator for envelope-tracking activity is more strongly shaped by bottom-up auditory processing than top-down feedback from consciousness-related cortical areas that are impaired by DoC.

## Synthesis

Reviewing Editor: Anne Keitel, University of Dundee Decisions are customarily a result of the Reviewing Editor and the peer reviewers coming together and discussing their recommendations until a consensus is reached. When revisions are invited, a fact-based synthesis statement explaining their decision and outlining what is needed to prepare a revision will be listed below. The following reviewer(s) agreed to reveal their identity: Sanne ten Oever.

The reviewers and editor agreed that this is an interesting and timely study topic. However, several issues were brought up by the reviewers, which are added below. The following main points were identified in joint discussions:

1) Elaborate on the underlying hypotheses and embed the manuscript further in previous research.

2) Phrase the results in a way that reflects the less strong responses in patients with DoC.

3) Flesh out the main messages and reasoning of the manuscript.

You will find these main issues as well as other comments in the unabridged reviewer comments below. Please address all comments in a point-by-point manner.

*****

Reviewer #1 - Advances the Field (Required)

The findings from the study inform speech-processing in DOC.

Reviewer #1 - Comments to the Authors (Required)

The authors provide a compelling study concisely written with advanced analyses and interesting findings providing important implications for the field of speech-processing and DOC. I think the study might even be stronger, if the background for the hypotheses was more elaborated and the implications of the findings more discussed.

The implications of not finding evidence for the presented hypothesis of modulations of the linear-phase property due to DOC,are in my view not fully discussed.

Although, I find the general line of argumentation and approach compelling, I would appreciate some more explanation for coming up with the hypothesis. From the introduction I got the impression (without it being explicitly stated), that the assumption is that bottom-up and top-down processes are differentially affected by DOC and that this might be measured by modifications of the linear-phase property.

In order to appreciate fully the implications of this study for the understanding of speech-processing in DOC, I would like the authors to clarify whether there is previous support for expecting influences on the linear-phase, how they expect the roles of top-down and bottom up in this scenario and relatedly how the findings from the different DOC groups might inform their speech-processing model.

Similarly, the question about underlying generator for the response phase is mainly approached from a very technical and mechanistic view (Oppenheim), but little is provided for the transfer of the actual neuronal generation. Two references are mentioned regarding “important information about how the envelope-tracking response is generated”, but there is very little further explanation on those important information beyond “envelope-tracking response shows the linear phase property and the response phase changes linearly as a function of frequency within the theta band”

Minor:

- Please add information about the delivery of the stimuli (loudspeaker, headphones, etc) and the surroundings (patient room, shielded room, etc)

- Page 7, paragraph 2.6. in the this sentence, the “If” seems misplaced.

- Page 7: strongest coherence in 14 electrodes is mentioned. I think it might help here to put a reference to the corresponding result section, as otherwise the reader might be asking at this moment, how and for which group those 14 electrodes were identified.

*****

Reviewer #2 - Advances the Field (Required)

I feel it show that in DoC patients there is some preserved auditory response. But this we already know. To me it is difficult from the current version of the manuscript to extract what the added new contribution is. It is not ERP work, but it shows the same preserved auditory response.

Reviewer #2 - Concerns about human or animal subjects

No ethics statement was provided for the newly collected data. It was not clear if the study went through any ethical board.

Reviewer #2 - Comments to the Authors (Required)

Review manuscript

In this manuscript the authors investigated speech tracking in healthy individuals as well as individuals with a disorder of consciousness. They found overall speech tracking as measured with phase coherence between the speech envelope and the EEG in all participants, but most strongly in the healthy subjects. In all participants there was a trend that there was a linear frequency-phase relationship.

Mayor comments:

- I think the authors make rather strong claims. They claim that they see preserved envelope tracking for all DoC patients. While for the EMCS and MCS this could be said, for the UWS that is clearly not the case as none of the effects are significant. Also, from the whole manuscript it sounds like the effects are almost identical in healthy subjects that in the DoC patients. While I agree that form EMCS and MCS there is preservation of the response, it is still clearly weaker as for the healthy participants and for some participants there is no phase coherence with the speech signal. I suggest that the authors are more careful in their phrasing.

- The author in their discussion correctly state that the most logical explanation of a linear phase-frequency relationship is simply that the response of the brain is some lagged form or at least a consistent lagged response to the input. But did we not already know that DoC patients respond to auditory stimuli? It might be spelled out a bit more clearly what the added value of the current manuscript is. What did we learn? Alternatively, it sounds that the authors would claim that in healthy subject phase tracking is only due to bottom-up effects as it is preserved in DoC patients? From the text this did not become fully clear to me what the main message is.

- The authors of state that effects are weaker when there is no phase consistency. This to me seems rather trivial. How could there be any systematic frequency-phase relationship when there was no systematic phase relationship to start with?

- While there seems to be some preserved linearity of the phase-frequency curve for the EMCS/MCS group, the group delay is not consistent across participants. I wonder what this indicates? Could it be that there is more variability in the latency of the response across these group simply because it is more heterogeneous or would this indicate something else?

- One conclusion in the discussion reads ‘It is demonstrated that the linear-phase property can be observed in both healthy individuals and in EMCS/MCS/UWS patients who exhibit reliable neural synchronization to speech, indicating the linearphase property is not strongly modulated by the state of consciousness’. In some sense the data shows this effect, but is it meaningful? If many patients don’t synchronize well, isn’t it more interesting to report that they don’t synchronize. This relates a bit to a general unclearness to what the point of the study is. Is it to show that the normal tracking response remain linear independent of DoC or to show that we can find speech tracking similar to healthy subjects in DoC patients?

Minor comment:

- The authors calculate the envelope by applying a full-wave rectification and low-pass filtering the data. I’m not sure if this reflects a similar envelope as what has bene done in previous speech tracking studies. Often one extracts the magnitude of the Hilbert transformed signal either of the whole signal or of separate bands. I’m not sure whether the transformation as done here will provide a similar estimate. I might be wrong, but it might be good to compare the methods.

- Why did the authors not do any tapering to their FFT analysis? It is possible that you will get some distortions in your FFT estimation due to not fulfilling the assumption of stationarity.

- I would think some discussion on how these effects relate to preserved perceptual functioning in DoC patients would be good. In some sense the authors find that there is a preserved response to auditory stimuli that mostly can be explained by bottom-up processing. I think this should be put in light of previous findings of responses of DoC patients to other stimuli, for example ERP work.

## References

[B1] Ashwal S (1994) The persistent vegetative state in children. Adv Pediatr 41:195–222. 7992684

[B2] Beukema S, Gonzalez-Lara LE, Finoia P, Kamau E, Allanson J, Chennu S, Gibson RM, Pickard JD, Owen AM, Cruse D (2016) A hierarchy of event-related potential markers of auditory processing in disorders of consciousness. Neuroimage Clin 12:359–371. 10.1016/j.nicl.2016.08.003 27595064 PMC4995605

[B3] Bourguignon M, Molinaro N, Lizarazu M, Taulu S, Jousmäki V, Lallier M, Carreiras M, De Tiège X (2020) Neocortical activity tracks the hierarchical linguistic structures of self-produced speech during reading aloud. Neuroimage 216:116788. 10.1016/j.neuroimage.2020.116788 32348908

[B4] Braiman C, Fridman EA, Conte MM, Voss HU, Reichenbach CS, Reichenbach T, Schiff ND (2018) Cortical response to the natural speech envelope correlates with neuroimaging evidence of cognition in severe brain injury. Curr Biol 28:3833–3839.e3. 10.1016/j.cub.2018.10.057 30471997

[B5] Brodbeck C, Hong LE, Simon JZ (2018) Rapid transformation from auditory to linguistic representations of continuous speech. Curr Biol 28:3976–3983.e5. 10.1016/j.cub.2018.10.042 30503620 PMC6339854

[B6] Crosse MJ, Di Liberto GM, Lalor EC (2016) Eye can hear clearly now: inverse effectiveness in natural audiovisual speech processing relies on long-term crossmodal temporal integration. J Neurosci 36:9888–9895. 10.1523/JNEUROSCI.1396-16.2016 27656026 PMC6705572

[B7] Daniel K, Christian KF, Vibe GF, Martin F, Kirsten M (2016) Preserved consciousness in vegetative and minimal conscious states: systematic review and meta-analysis. J Neurol Neurosurg Psychiatry 87:485. 26139551 10.1136/jnnp-2015-310958

[B8] Ding N, Simon JZ (2012a) Neural coding of continuous speech in auditory cortex during monaural and dichotic listening. J Neurophysiol 107:78–89. 10.1152/jn.00297.2011 21975452 PMC3570829

[B9] Ding N, Simon JZ (2012b) Emergence of neural encoding of auditory objects while listening to competing speakers. Proc Natl Acad Sci U S A 109:11854–11859. 10.1073/pnas.1205381109 22753470 PMC3406818

[B10] Ding N, Patel AD, Chen L, Butler H, Luo C, Poeppel D (2017) Temporal modulations in speech and music. Neurosci Biobehav Rev 81:181–187. 10.1016/j.neubiorev.2017.02.011 28212857

[B11] Ding N, Pan X, Luo C, Su N, Zhang W, Zhang J (2018) Attention is required for knowledge-based sequential grouping: insights from the integration of syllables into words. J Neurosci 38:1178–1188. 10.1523/JNEUROSCI.2606-17.2017 29255005 PMC6596269

[B12] Doelling KB, Arnal LH, Ghitza O, Poeppel D (2014) Acoustic landmarks drive delta-theta oscillations to enable speech comprehension by facilitating perceptual parsing. Neuroimage 85 [Pt 2]:761–768. 10.1016/j.neuroimage.2013.06.035 23791839 PMC3839250

[B13] Doelling KB, Assaneo MF, Bevilacqua D, Pesaran B, Poeppel D (2019) An oscillator model better predicts cortical entrainment to music. Proc Natl Acad Sci U S A 116:10113–10121. 10.1073/pnas.1816414116 31019082 PMC6525506

[B14] Drullman R, Festen JM, Plomp R (1994) Effect of temporal envelope smearing on speech reception. J Acoust Soc Am 95:2670– 2680. 10.1121/1.409836 8132899

[B15] Elliott TM, Theunissen FE (2009) The modulation transfer function for speech intelligibility. PLoS Comput Biol 5:e1000302. 10.1371/journal.pcbi.1000302 19266016 PMC2639724

[B16] Fischer C, Morlet D, Giard M-H (2000) Mismatch negativity and N100 in comatose patients. Audiol Neurootol 5:192–197. 10.1159/000013880 10859413

[B17] Fisher NI (1993) Statistical analysis of circular data. Cambridge: Cambridge University Press.

[B18] Giacino JT, Ashwal S, Childs N, Cranford R, Jennett B, Katz DI, Kelly JP, Rosenberg JH, Whyte J, Zafonte RD, Zasler ND (2002) The minimally conscious state: definition and diagnostic criteria. Neurology 58:349–353. 10.1212/wnl.58.3.349 11839831

[B19] Giacino JT, Fins JJ, Laureys S, Schiff ND (2014) Disorders of consciousness after acquired brain injury: the state of the science. Nat Rev Neurol 10:99–114. 10.1038/nrneurol.2013.279 24468878

[B20] Giacino JT, Katz DI, Schiff ND, Whyte J, Ashman EJ, Ashwal S, Barbano R, Hammond FM, Laureys S, Ling GSF, Nakase-Richardson R, Seel RT, Yablon S, Getchius TSD, Gronseth GS, Armstrong MJ (2018) Practice guideline update recommendations summary: disorders of consciousness: report of the guideline development, dissemination, and implementation subcommittee of the American Academy of Neurology; the American Congress of Rehabilitation Medicine; and the National Institute on Disability, Independent Living, and Rehabilitation Research. Archives of Physical Medicine and Rehabilitation 91:450– 460. 10.1212/WNL.0000000000005926 30089618 PMC6139814

[B21] Giordano BL, Ince RAA, Gross J, Schyns PG, Panzeri S, Kayser C (2017) Contributions of local speech encoding and functional connectivity to audio-visual speech perception. Elife 6:e24763. 10.7554/eLife.24763 28590903 PMC5462535

[B22] Gui P, Jiang Y, Zang D, Qi Z, Tan J, Tanigawa H, Jiang J, Wen Y, Xu L, Zhao J, Mao Y, Poo M-m, Ding N, Dehaene S, Wu X, Wang L (2020) Assessing the depth of language processing in patients with disorders of consciousness. Nat Neurosci 23:761–770. 10.1038/s41593-020-0639-1 32451482

[B23] Harding EE, Sammler D, Henry MJ, Large EW, Kotz SA (2019) Cortical tracking of rhythm in music and speech. Neuroimage 185:96–101. 10.1016/j.neuroimage.2018.10.037 30336253

[B24] Henry MJ, Obleser J (2012) Frequency modulation entrains slow neural oscillations and optimizes human listening behavior. Proc Natl Acad Sci U S A 109:20095–20100. 10.1073/pnas.1213390109 23151506 PMC3523826

[B25] Jeremy DT, Maarten De V, Filipa Campos V, Stefan D (2011) Cross-modal phase reset predicts auditory task performance in humans. J Neurosci 31:3853–3861. 21389240 10.1523/JNEUROSCI.6176-10.2011 PMC6622791

[B26] Kong YY, Mullangi A, Ding N (2014) Differential modulation of auditory responses to attended and unattended speech in different listening conditions. Hear Res 316:73–81. 10.1016/j.heares.2014.07.009 25124153 PMC4194271

[B27] Koskinen M, Seppä M (2014) Uncovering cortical MEG responses to listened audiobook stories. Neuroimage 100:263–270. 10.1016/j.neuroimage.2014.06.018 24945666

[B28] Lakatos P, Musacchia G, O’Connel MN, Falchier AY, Javitt DC, Schroeder CE (2013) The spectrotemporal filter mechanism of auditory selective attention. Neuron 77:750–761. 10.1016/j.neuron.2012.11.034 23439126 PMC3583016

[B29] Lalor EC, Power AJ, Reilly RB, Foxe JJ (2009) Resolving precise temporal processing properties of the auditory system using continuous stimuli. J Neurophysiol 102:349–359. 10.1152/jn.90896.2008 19439675

[B30] Laureys S, Celesia GG, Cohadon F, Lavrijsen J, León-Carrión J, Sannita WG, Sazbon L, Schmutzhard E, von Wild KR, Zeman A, Dolce G; European Task Force on Disorders of Consciousness (2010) Unresponsive wakefulness syndrome: a new name for the vegetative state or apallic syndrome. BMC Med 8:68. 10.1186/1741-7015-8-68 21040571 PMC2987895

[B31] Lu L, Deng Y, Xiao Z, Jiang R, Gao JH (2023) Neural signatures of hierarchical linguistic structures in second language listening comprehension. eNeuro 10:ENEURO.0346-0322.2023. 10.1523/ENEURO.0346-22.2023 PMC10294774 37328296

[B32] Luo H, Poeppel D (2007) Phase patterns of neuronal responses reliably discriminate speech in human auditory cortex. Neuron 54:1001–1010. 10.1016/j.neuron.2007.06.004 17582338 PMC2703451

[B33] Mesgarani N, Chang EF (2012) Selective cortical representation of attended speaker in multi-talker speech perception. Nature 485:233–236. 10.1038/nature11020 22522927 PMC3870007

[B34] Ng BSW, Schroeder T, Kayser C (2012) A precluding but not ensuring role of entrained low-frequency oscillations for auditory perception. J Neurosci 32:12268–12276. 10.1523/JNEUROSCI.1877-12.2012 22933808 PMC6621531

[B35] Oppenheim AV, Willsky AS, Nawab SH (1997) Signals and systems. Hoboken: Prentice Hall.

[B36] O’Sullivan JA, Power AJ, Mesgarani N, Rajaram S, Foxe JJ, Shinn-Cunningham BG, Slaney M, Shamma SA, Lalor EC (2015) Attentional selection in a cocktail party environment can be decoded from single-trial EEG. Cereb Cortex 25:1697–1706. 10.1093/cercor/bht355 24429136 PMC4481604

[B37] Peelle JE, Gross J, Davis MH (2013) Phase-locked responses to speech in human auditory cortex are enhanced during comprehension. Cereb Cortex 23:1378–1387. 10.1093/cercor/bhs118 22610394 PMC3643716

[B38] Power AJ, Foxe JJ, Forde EJ, Reilly RB, Lalor EC (2012) At what time is the cocktail party? A late locus of selective attention to natural speech. Eur J Neurosci 35:1497–1503. 10.1111/j.1460-9568.2012.08060.x 22462504

[B39] Qin P, Di H, Yan X, Yu S, Yu D, Laureys S, Weng X (2008) Mismatch negativity to the patient’s own name in chronic disorders of consciousness. Neurosci Lett 448:24–28. 10.1016/j.neulet.2008.10.029 18938213

[B40] Schroeder CE, Lakatos P, Kajikawa Y, Partan S, Puce A (2008) Neuronal oscillations and visual amplification of speech. Trends Cogn Sci 12:106–113. 10.1016/j.tics.2008.01.002 18280772 PMC3987824

[B41] Shamma S (2001) On the role of space and time in auditory processing. Trends Cogn Sci 5:340–348. 10.1016/s1364-6613(00)01704-6 11477003

[B42] Shannon RV, Zeng FG, Kamath V, Wygonski J, Ekelid M (1995) Speech recognition with primarily temporal cues. Science 270:303–304. 10.1126/science.270.5234.303 7569981

[B43] Sharpee TO, Atencio CA, Schreiner CE (2011) Hierarchical representations in the auditory cortex. Curr Opin Neurobiol 21:761–767. 10.1016/j.conb.2011.05.027 21704508 PMC3223290

[B44] Steinschneider M, Nourski KV, Fishman YI (2013) Representation of speech in human auditory cortex: is it special? Hear Res 305:57–73. 10.1016/j.heares.2013.05.013 23792076 PMC3818517

[B45] van Atteveldt N, Murray MM, Thut G, Schroeder CE (2014) Multisensory integration: flexible use of general operations. Neuron 81:1240–1253. 10.1016/j.neuron.2014.02.044 24656248 PMC4090761

[B46] Wang Y, Ding N, Ahmar N, Xiang J, Poeppel D, Simon JZ (2012) Sensitivity to temporal modulation rate and spectral bandwidth in the human auditory system: MEG evidence. J Neurophysiol 107:2033–2041. 10.1152/jn.00310.2011 21975451 PMC3331605

[B47] Wang Y, Zhang J, Zou J, Luo H, Ding N (2019) Prior knowledge guides speech segregation in human auditory cortex. Cereb Cortex 29:1561–1571. 10.1093/cercor/bhy052 29788144

[B48] Xu C, Zou J, He F, Wen X, Li J, Gao J, Ding N, Luo B (2021) Neural tracking of sound rhythms correlates with diagnosis, severity, and prognosis of disorders of consciousness. Front Neurosci 15:646543. 10.3389/fnins.2021.646543 33994924 PMC8113690

[B49] Xu C, Li H, Gao J, Li L, He F, Yu J, Ling Y, Gao J, Li J, Melloni L, Luo B, Ding N (2023) Statistical learning in patients in the minimally conscious state. Cereb Cortex 33:2507–2516. 10.1093/cercor/bhac222 35670595

[B50] Yang X, Wang K, Shamma SA (1992) Auditory representations of acoustic signals. IEEE Trans Inform Theory 38:824–839. 10.1109/18.119739

[B51] Zion Golumbic E, Cogan GB, Schroeder CE, Poeppel D (2013a) Visual input enhances selective speech envelope tracking in auditory cortex at a “cocktail party.” J Neurosci 33:1417–1426. 10.1523/JNEUROSCI.3675-12.2013 PMC3711546 23345218

[B52] Zion Golumbic EM, Ding N, Bickel S, Lakatos P, Schevon CA, McKhann GM, Goodman RR, Emerson R, Mehta AD, Simon JZ, Poeppel D, Schroeder CE (2013b) Mechanisms underlying selective neuronal tracking of attended speech at a “cocktail party.” Neuron 77:980–991. 10.1016/j.neuron.2012.12.037 PMC3891478 23473326

[B53] Zou J, Feng J, Xu T, Jin P, Luo C, Zhang J, Pan X, Chen F, Zheng J, Ding N (2019) Auditory and language contributions to neural encoding of speech features in noisy environments. Neuroimage 192:66–75. 10.1016/j.neuroimage.2019.02.047 30822469

[B54] Zou J, Xu C, Luo C, Jin P, Gao J, Li J, Gao J, Ding N, Luo B (2021) θ-Band cortical tracking of the speech envelope shows the linear phase property. eNeuro 8:ENEURO.0058-21.2021. 10.1523/ENEURO.0058-21.2021 PMC8387159 34380659

